# Supplementary material for: Stillbirth incidence and determinants in a tertiary health facility in the Volta Region of Ghana
Source: PLoS One. 2023 Dec 21;18(12):e0296076. doi: 10.1371/journal.pone.0296076 (PMC10734929; doi:10.1371/journal.pone.0296076)
Supplement: S1 Checklist — (DOCX) [file pone.0296076.s001.docx]

STROBE Statement—checklist of items that should be included in reports of observational studies

|  | Item No. | Recommendation | Page  No. | Relevant text from manuscript |
| --- | --- | --- | --- | --- |
| **Title and abstract** | 1 | (*a*) Indicate the study’s design with a commonly used term in the title or the abstract | Page 2 (abstract/methods) | **This was a prospective cohort study involving pregnant women admitted for delivery at HTH between October 2019 and March 2020………..** |
|  |  | (*b*) Provide in the abstract an informative and balanced summary of what was done and what was found | Page 2 |  |
| Introduction | | | |  |
| Background/rationale | 2 | Explain the scientific background and rationale for the investigation being reported | **Pages 3 and 4** |  |
| Objectives | 3 | State specific objectives, including any prespecified hypotheses | **Page 4** | **It is therefore pertinent to assess current stillbirth rates and associating risk factors in a prospective study to enable health managers and policy makers evaluate the effectiveness of existing interventions including the quality of obstetric care and the need for new interventions based on newly found dynamics.** |
| Methods | | | |  |
| Study design | 4 | Present key elements of study design early in the paper Pages 2 and 5 |  |  |
| Setting | 5 | Describe the setting, locations, and relevant dates, including periods of recruitment, exposure, follow-up, and data collection | **Pages 5 and 6** |  |
| Participants | 6 | (*a*) *Cohort study*—Give the eligibility criteria, and the sources and methods of selection of participants. Describe methods of follow-up **Page 6**  *Case-control study*—Give the eligibility criteria, and the sources and methods of case ascertainment and control selection. Give the rationale for the choice of cases and controls  *Cross-sectional study*—Give the eligibility criteria, and the sources and methods of selection of participants |  | **Women, referred and non-referred, admitted for labour and delivery or for prelabour caesarean section at HTH within the study period together with their new born babies were eligible to participate in the study. The women referred with intrauterine fetal deaths (IUFD) and those that delivered live or dead babies before 28 completed weeks of gestation (abortions) at HTH were excluded.** |
|  |  | (*b*) *Cohort study*—For matched studies, give matching criteria and number of exposed and unexposed  *Case-control study*—For matched studies, give matching criteria and the number of controls per case |  |  |
| Variables | 7 | Clearly define all outcomes, exposures, predictors, potential confounders, and effect modifiers. Give diagnostic criteria, if applicable | **Pages 6 and 7** |  |
| Data sources/ measurement | 8* | For each variable of interest, give sources of data and details of methods of assessment (measurement). Describe comparability of assessment methods if there is more than one group | **Pages 6-8** |  |
| Bias | 9 | Describe any efforts to address potential sources of bias | **Page 6** | **Both singleton and twin pregnancies were included.**  **…..The women referred with intrauterine fetal deaths (IUFD) and those that delivered live or dead babies before 28 completed weeks of gestation (abortions) at HTH were excluded.** |
| Study size | 10 | Explain how the study size was arrived at **Page 5** |  | **There was no formal sample size estimation for this study. All women admitted for labour and delivery or prelabour caesarean section and their new born babies, over the study period, were assessed for eligibility and included if found eligible and if consent was given. In all, 696 women (with their 714 newborns) were assessed for study inclusion. Of these, 687 women (and 702 newborns) were found eligible and included in the study………** |

Continued on next page

| Quantitative variables | 11 | Explain how quantitative variables were handled in the analyses. If applicable, describe which groupings were chosen and why | **Page 7** | **……. Descriptive analyses were performed and presented as frequencies, percentages, means and standard deviation**  **…… Univariate logistic regression analysis was used to assess for associations between stillbirths and the independent variables including participant’s socio-demographic characteristics and obstetric factors of the index pregnancy. Those with p-values <0.05 were entered into a multivariate model to arrive at the final regression model. Odds ratios and their 95% confidence intervals were reported. An association in the final multivariate logistic regression model was considered statistically significant if p <0.05.** |
| --- | --- | --- | --- | --- |
| Statistical methods | 12 | (*a*) Describe all statistical methods, including those used to control for confounding Page 7 |  | **……. Descriptive analyses were performed and presented as frequencies, percentages, means and standard deviation**  **…… Univariate logistic regression analysis was used to assess for associations between stillbirths and the independent variables including participant’s socio-demographic characteristics and obstetric factors of the index pregnancy. Those with p-values <0.05 were entered into a multivariate model to arrive at the final regression model. Odds ratios and their 95% confidence intervals were reported. An association in the final multivariate logistic regression model was considered statistically significant if p <0.05.** |
|  |  | (*b*) Describe any methods used to examine subgroups and interactions |  |  |
|  |  | (*c*) Explain how missing data were addressed |  | All efforts were made to get data on all requisite variables while the study women were still on admission in the hospital |
|  |  | (*d*) *Cohort study*—If applicable, explain how loss to follow-up was addressed  *Case-control study*—If applicable, explain how matching of cases and controls was addressed  *Cross-sectional study*—If applicable, describe analytical methods taking account of sampling strategy |  | There were no losses to follow-up in this study as the follow-up done was over admission in the hospital until delivery |
|  |  | (*e*) Describe any sensitivity analyses |  |  |
| Results | | | | |
| Participants | 13* | (a) Report numbers of individuals at each stage of study—eg numbers potentially eligible, examined for eligibility, confirmed eligible, included in the study, completing follow-up, and analysed | **Page 5 and 9** | **Of 696 pregnant women assessed for inclusion, 9 were excluded (5 were referred with IUFD and 4 delivered before 28 weeks gestation). Six hundred and eighty-seven (687) women with their 702 newborns thus participated in the study and contributed data for analysis.** |
|  |  | (b) Give reasons for non-participation at each stage | **Page 9** |  |
|  |  | (c) Consider use of a flow diagram |  |  |
| Descriptive data | 14* | (a) Give characteristics of study participants (eg demographic, clinical, social) and information on exposures and potential confounders | **Pages 9-11** |  |
|  |  | (b) Indicate number of participants with missing data for each variable of interest |  |  |
|  |  | (c) *Cohort study*—Summarise follow-up time (eg, average and total amount) |  |  |
| Outcome data | 15* | *Cohort study*—Report numbers of outcome events or summary measures over time | **Page 12** |  |
|  |  | *Case-control study—*Report numbers in each exposure category, or summary measures of exposure |  |  |
|  |  | *Cross-sectional study—*Report numbers of outcome events or summary measures |  |  |
| Main results | 16 | (*a*) Give unadjusted estimates and, if applicable, confounder-adjusted estimates and their precision (eg, 95% confidence interval). Make clear which confounders were adjusted for and why they were included | **Pages 13-15** | **Table 3** |
|  |  | (*b*) Report category boundaries when continuous variables were categorized |  |  |
|  |  | (*c*) If relevant, consider translating estimates of relative risk into absolute risk for a meaningful time period |  |  |

Continued on next page

| Other analyses | 17 | Report other analyses done—eg analyses of subgroups and interactions, and sensitivity analyses |  |  |
| --- | --- | --- | --- | --- |
| Discussion | | | | |
| Key results | 18 | Summarise key results with reference to study objectives **Page 16** |  | **The study assessed the incidence and factors influencing stillbirths among deliveries at Ho Teaching Hospital. An overall stillbirth incidence rate of 31.3 per 1000 births was observed in the study with preterm delivery at 28 – 34 weeks’ gestation and labour induction as key risk factors. Among pregnancies referred to HTH for delivery, the stillbirth incidence was 43 per 1000 births while among women with hypertensive disorders of pregnancy, it was 114 per 1000 births. The prevalence of hypertensive disorders of pregnancy was 9.8% and preeclampsia and chronic hypertension in pregnancy were the most and the least common forms respectively. To the best of the authors’ knowledge, this is the first study reporting on stillbirth incidence and a comprehensive array of risk factors in the Volta Region. The study provides vital baseline information for action to alleviate the burden of stillbirths in HTH and possibly the rest of the Volta Region.............** |
| Limitations | 19 | Discuss limitations of the study, taking into account sources of potential bias or imprecision. Discuss both direction and magnitude of any potential bias | **Page 22** | **…..However, the 95% CI around the odds for the gestational age, labour induction and birthweight were wide. While these may suggest a low level of precision for the associations with stillbirths and the need for cautious interpretation of these particular findings, it must be noted that the wide intervals arise mostly from the small sizes of the sub-categories under these variables. Furthermore, the study was done at a single site and the findings thereof may have limited external validity. Nevertheless, the study reports findings comparable to previous ones and provide fine scale data for targeted action to reduce stillbirths.** |
| Interpretation | 20 | Give a cautious overall interpretation of results considering objectives, limitations, multiplicity of analyses, results from similar studies, and other relevant evidence | **Page 22** |  |
| Generalisability | 21 | Discuss the generalisability (external validity) of the study results | **Page 22** |  |
| Other information | |  | | |
| Funding | 22 | Give the source of funding and the role of the funders for the present study and, if applicable, for the original study on which the present article is based |  | No funding for this study…..included during submission |

*Give information separately for cases and controls in case-control studies and, if applicable, for exposed and unexposed groups in cohort and cross-sectional studies.

**Note:** An Explanation and Elaboration article discusses each checklist item and gives methodological background and published examples of transparent reporting. The STROBE checklist is best used in conjunction with this article (freely available on the Web sites of PLoS Medicine at http://www.plosmedicine.org/, Annals of Internal Medicine at http://www.annals.org/, and Epidemiology at http://www.epidem.com/). Information on the STROBE Initiative is available at www.strobe-statement.org.
